# Comparison between a flash glucose monitoring system and a portable blood glucose meter for monitoring dogs with diabetes mellitus

**DOI:** 10.1111/jvim.15930

**Published:** 2020-10-30

**Authors:** Francesca Del Baldo, Claudia Canton, Silvia Testa, Harry Swales, Ignazio Drudi, Stefania Golinelli, Federico Fracassi

**Affiliations:** ^1^ Department of Veterinary Medical Science University of Bologna, Ozzano dell'Emilia Bologna Italy; ^2^ Small Animal Teaching Hospital, Leahurst Campus University of Liverpool Wirral United Kingdom; ^3^ Department of Statistical Sciences University of Bologna Bologna Italy

**Keywords:** canine, diabetes mellitus, FreeStyle Libre, interstitial glucose

## Abstract

**Background:**

Flash glucose monitoring system (FGMS; FreeStyle Libre) was recently validated for use in diabetic dogs (DD). It is not known if this system is clinically useful in monitoring DD.

**Objective:**

To compare the clinical utility of FGMS against blood glucose curves (BGCs) obtained with a portable blood glucose meter (PBGM) in monitoring DD.

**Animals:**

Twenty dogs with diabetes mellitus.

**Methods:**

Prospective study. Dogs with diabetes mellitus on insulin treatment for at least 1 month were included. Comparisons of insulin dose recommendations based on the in‐hospital GCs acquired using FGMS and a PBGM, consecutive‐day interstitial GCs (IGCs) acquired at home using the FGMS, and consecutive‐day, home vs hospital IGCs acquired using the FGMS were made using concordance analysis.

**Results:**

There was good concordance between insulin dose recommendations based on FGMS and PBGM generated GCs and IGCs obtained in the 2 different environments on 2 consecutive days, but almost absent concordance between IGCs obtained on 2 consecutive days at home. Glucose nadirs were detected in 34/43 (79%) of Ambulatory Glucose Profile (AGP) reports of the FGMS. In comparison, concordant glucose nadirs were identified in 14/34 (41%) BGCs using PBGM. The individual FGMS scans and PBGM identified 60% and 9% of low IG/hypoglycemic episodes, respectively.

**Conclusions and Clinical Importance:**

Insulin dose adjustments based on BGCs can be suboptimal. The FGMS allows a more accurate identification of the glucose nadirs and hypoglycemic episodes compared to the use of a PBGM and assessment of day‐to‐day variations in glycemic control.

**Abbreviations**
AGP: ambulatory glucose profile
BGC: blood glucose curve
CGMS: continuous glucose monitoring system
DM: diabetes mellitus
FGMS: flash glucose monitoring system
IG: interstitial glucose
PBGM: portable blood glucose meter

## 1 INTRODUCTION

Diabetes mellitus (DM) is a common endocrine disease of dogs characterized by an absolute or relative deficiency of insulin.^1^ Dogs with DM are treated with exogenous insulin and require regular monitoring to ensure appropriate dosing. Tools available to veterinarians for monitoring the response of diabetic dogs to treatment include clinical signs, body weight, glycated proteins levels, and blood glucose curves (BGCs) among others.^1^ Typically, BGCs are conducted in a hospital setting or at home and involve 1‐to‐2 hourly blood sampling with a portable blood glucose meter (PBGM) over an 8 to 12 hour period. Evaluation of BGCs allows clinicians to determine glucose nadir, time to nadir, mean blood glucose concentration as well as assessing the degree of variation in blood glucose concentration.^1^ This method has some disadvantages such as the need for repeated venipuncture, that can be stressful and painful for the animal, but also carries the risk of missing the blood glucose peak or nadir if they fall between 2 sampling times.^2^Additionally, in‐hospital BGCs are time consuming, expensive and do not allow the assessment of glycemia on consecutive days. This last aspect represents an important limitation because of the variability of serial blood glucose curves in dogs and humans.^3^, ^4^

Continuous glucose monitoring systems (CGMS) are used routinely in human diabetic patients and several studies have demonstrated their accuracy and clinical utility in veterinary medicine.^2^, ^5^, ^6^, ^7^, ^8^, ^9^, ^10^, ^11^ CGMS typically consist of a sensor which measures interstitial glucose (IG) and relays the recorded measurements to a transmitter. The devices used in most previous veterinary studies have a number of limitations not limited to the need for frequent calibration with circulating blood glucose, but also having a limited monitoring period.

Recently, a novel flash glucose monitoring system (FGMS) has been licensed for the use in a number of countries. The device measures IG levels on a minute‐by‐minute basis via a disc shaped sensor with a small catheter inserted under the skin that can record measurements for up to 14 days. In contrast to other CGMS, this FGMS does not require calibration and the device is accurate when evaluating IG in dogs with diabetes mellitus and is well tolerated by dogs.^11^ However, studies evaluating the clinical use of FGMS in the monitoring of dogs with DM are lacking. Furthermore, whether treatment decisions based on GCs obtained with the FGMS differ from those derived using a PBGM has not been investigated in dogs. Therefore, the aims of the present study were (a) to compare the recommended insulin dose based on the evaluation of IGCs obtained by the FGMS with BGCs obtained by a PBGM; (b) to compare the recommended insulin dose based on evaluation of IGCs obtained by the FGMS on 2 consecutive days both in the same environment and in different environments (hospital and home); (c) to compare the ability of the FGMS to detect glucose nadir as well low IG episodes against BGCs obtained by a PBGM; and (d) to compare day‐time and night‐time glucose nadirs obtained by the use of FGMS.

## 2 MATERIALS AND METHODS

### 2.1 Dogs with diabetes mellitus

Twenty client‐owned dogs with diabetes mellitus and admitted to the Veterinary University Hospital between May 2015 and March 2018 were prospectively enrolled into the study.

DM was diagnosed based on consistent clinical signs including polyuria, polydipsia, weakness and weight loss alongside a blood glucose concentration >180 mg/dL (>11 mmol/L) after food had been withheld for at least 10 hours, glucosuria and serum fructosamine concentration >340 μmol/L (reference interval: 222‐382 μmol/L).^12^

All dogs had been treated with insulin for at least 4 weeks prior to enrolment in the study. Owners provided written informed consent for inclusion of their dogs in the study. The study was approved by the local Scientific Ethics Committee for Animal Testing.

### 2.2 Flash glucose monitoring system

The FreeStyle Libre Flash Glucose Monitoring System (FGMS) was used in this study and is composed of a small, lightweight disc‐shaped sensor (35 mm × 5 mm). The sensor measures the IG concentration through a small, subcutaneous catheter (0.4 mm × 5 mm). Glucose detection is based on Wired Enzyme Technology, that consists of both enzymatic (glucose oxidase) and amperometric (electrodes) systems.^13^ Reduction of glucose by glucose oxidase results in generation of an electric current, the intensity of which is proportional to the IG concentration.

**FIGURE 1:**
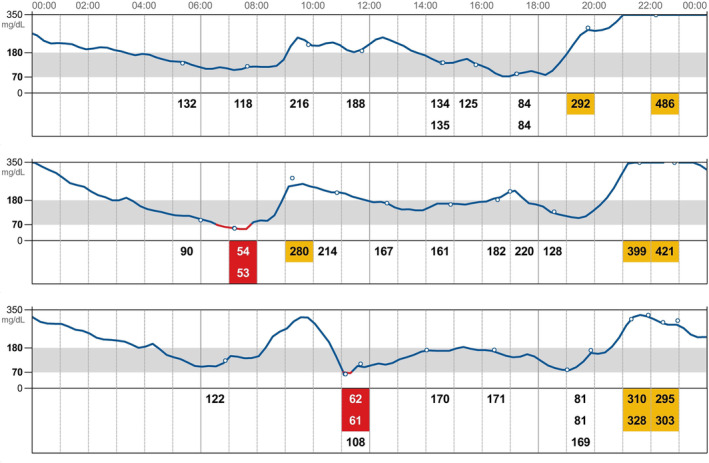
Daily log report showing IG fluctuations during a 24 hours period. Interstitial glucose values detected by the scans are reported as numbers and are identified by the empty circles. Red box highlight IG values <70 mg/dL while yellow box highlight IG values >350 mg/dL. Low IG episodes and glucose nadirs extracted from the daily log report of each patient were used as the “gold standard” in order to identify the ability of the PBGM to detect glucose nadirs and low IG/hypoglycemic episodes. IG, interstitial glucose; PBGM, portable blood glucose meter

The detection limits of the sensor are between 20 and 500 mg/dL and measurements outside of this range are recorded as “LO” and “HI,” respectively. The system is factory‐calibrated and consequently does not require calibration before or during the wearing period. The sensor begins recording data 1 hour after its application and automatically measures the IG concentration every minute. IG concentrations are transferred from the sensor to a reader when the user brings the hand‐held reader into close proximity to the sensor. The hand‐held reader then displays the current sensor IG concentration, an IG trend arrow, as well as IG concentrations over the preceding 8 hours. Scanning can be performed as often as is needed for current IG concentration, otherwise the measurements are automatically recorded and stored on the sensor (every 15 minutes) and displayed on the reader when scanned. The reader stores data for 90 days. Data can be uploaded from the reader, using the Abbott FreeStyle Libre software to generate summary glucose reports (Ambulatory Glucose Profile, AGP). Among these, the daily log report shows IG fluctuations between 20 and 350 mg/dL during a 24‐hour period (Figure 1) and, as such, it was used in this study to provide a rough estimation of the glucose nadirs as well as the number of low glucose episodes. At the end of recording period, the sensor is fully disposable, but the reader can be reused for a new sensor. The sensor was applied as previously described^11^ on the dorsal aspect of the neck.

Blood glucose measurements were acquired with a validated PBGM^14^ (Optium Xceed, Abbott Laboratories, Witney, England).

### 2.3 Timing

Seven separate GCs were acquired for each dog during the recording period of their respective FGMS (Table 1). During the recording period, each day was divided into 2‐time intervals: day‐time and night‐time. Day‐time was approximately defined as the time interval between the morning insulin administration and the evening insulin administration (08:00‐20:00). Night‐time was approximately defined as the time interval between the evening insulin administration and the next morning insulin administration (20:00‐08:00). On days 1, 7, and 14, paired, in‐hospital GCs were acquired using the FGMS and PBGM devices. On days 5, 6, 12, and 13, home IGCs were acquired using only the FGMS device by the dogs' respective owners. On day 1 of the study, dogs were hospitalized and the sensor was applied. For a total of 10 to 12 hours, IG glucose measurements were recorded using the FGMS on a 2‐hourly basis. Capillary blood glucose was obtained from the pinna every 2 hours using the PBGM during the same period. On days 7 and 14, food and insulin were given at home and the paired GCs were started after the dog arrived at the clinic (≤1 hour after insulin administration) using the same protocol. On the remaining days, owners acquired IGCs every 1 to 2 hours during the day‐time using the FGMS—recording displayed values in a diary.

**TABLE 1:** Timing of the study

Day	Environment	Glucose curves (GCs)
1	Hospital	Paired GCs with both FGMS and PBGM
5	Home	Single IGC with FGMS
6	Home	Single IGC with FGMS
7	Hospital	Paired GCs with both FGMS and PBGM
12	Home	Single IGC with FGMS
13	Home	Single IGC with FGMS
14	Hospital	Paired GCs with both FGMS and PBGM

Abbreviations: FGMS, flash glucose monitoring system; GCs, glucose curves; IGC, interstitial glucose curve; PBGM, portable blood glucose meter.

At the end of the recording period, the sensor was removed and the FGMS data were downloaded onto a personal computer using the Abbott FreeStyle Libre Software.

### 2.4 Assessment of IGCs and BGCs

Based on assessment of the GCs, for each GC, 2 hypothetical insulin dose recommendations were made with the aim to maintain either more than 50% of BG/IG values between 90 and 250 mg/dL or BG/IG nadir between 90 and 180 mg/dL^15^ (Table 2).

**TABLE 2:** Variables used to define the adjustment of insulin therapy

Insulin dosage	% glycemic values (GV)	Glucose Nadir (GN)
Unchanged (↔)	At least 50% GV between 90 and 250 mg/dL	GN between 90 and 180 mg/dL
Increased (↑)	At least 50% GV > 250 mg/dL	GN > 180 mg/dL
Decreased (↓)	At least 50% GV < 90 mg/dL	GN < 90 mg/dL

Abbreviations: GN, glucose nadir; GV, glycemic values.

Comparison of insulin dose recommendations based on the in‐hospital GCs acquired using FGMS and PBGM was made on days 1, 7, and 14 (study aim 1). Comparison of insulin dose recommendations based on consecutive‐day IGCs acquired at home using the FGMS was made on days 5 and 6 as well as days 12 and 13 (study aim 2). Comparison of insulin dose recommendations based on consecutive‐day, home vs hospital IGCs acquired using the FGMS was made on days 6 and 7 as well as days 13 and 14 (study aim 3).

### 2.5 Assessment of nadirs

Glucose nadir was defined as the lowest glucose result during the day‐time period. Nadirs extracted from the “daily log report” of the AGP, those scanned by the FGMS reader and those detected by the PBGM on days 1, 7, and 14 were compared (study aim 3) (Figure 1). The nadirs extracted from the AGP software were considered concordant with those obtained by the FGMS scans and by the PBGM if they had the same time interval (±60 minutes), from the morning injection of insulin.

### 2.6 Comparison between day‐time and night‐time nadirs

The day‐time and night‐time nadirs, extracted from the daily log report of the AGP were compared (study aim 4) and were considered concordant if they fell within the same glycemic range: <90, 90‐180, and >180 mg/dL.

### 2.7 Assessment of hypoglycemic episodes

The number and duration of low IG values (<70 mg/dL) were recorded from the AGP software and were compared with the FGMS and PBGM recordings (study aim 3).

### 2.8 Data analysis

Statistical analysis was performed with the aid of 2 commercially available software (GraphPad Prism 7, Cran R statistical package). Normality was assessed using D'Agostino and Pearson tests and parametric or nonparametric tests were used accordingly. Nonnormal data were reported as median and ranges while normal data were expressed as mean ± SD.

In order to compare results of different combinations of factors (in‐hospital GCs acquired using FGMS and PBGM; consecutive‐day IGCs acquired at home using the FGMS; home vs hospital IGCs acquired using the FGMS) and considering the impossibility to define a “gold standard” the so‐called concordance analysis was used. Such a concordance has been defined in terms of decision about the insulin dose, coded as −1, 0, 1 (decrease, steady, increase). Even if a “gold standard” is not defined, this study aimed to investigate the reliability of an alternative (and much easier to use) tool of analysis, so we adopted the FGMS as the reference for PBGM performances when evaluating hypoglycemic episodes and glucose nadirs. Considered in this framework, our study is a “reliability” study, in the sense of concordance of diagnoses. A lot of measures of concordance are available in statistics, but considering our experimental framework consisting in multimodal qualitative variables, and the above statement about “reliability,” following the bio‐medical^16^ and the statistical literature,^17^ 4 indices^17^ have been calculated: Cohen's K, the intraclass correlation index, the polychoric correlation (all ranging from −1 to 1) and the Fisher's exact test for count data. The first 3 indices measure the correlation (ie, concordance) of 2 factors, the last tests the significance of differences in the outputs of the factors.

In order to compare day‐time and night‐time nadirs Wilcoxon test was used.

## 3 RESULTS

### 3.1 Diabetic dogs

There were 10 mixed‐breeds, 5 English Setters, 1 Springer Spaniel, 1 Yugoslavian Shepherd dog, 1 Pinscher, 1 Maltese, and 1 Poodle. Of these, 13 dogs were neutered females, 5 neutered males, and 2 entire males. The median age was 11 years and 1 month **(7** years and 2 months‐13 years and 8 months), the median body weight was 6.5 kg (range, 6‐64.1 kg) and median BCS was 5/9 (3/9‐8/9). Median time from the diagnosis of DM was 7.5 months (1‐59 months). Thirteen dogs were treated with porcine insulin zinc suspension (Caninsulin, MSD, Boxmeer, Netherlands), 5 with Neutral Protamine Hagedorn (NPH) human analogue insulin (Humulin I, Eli Lilly Italia S.p.A., Sesto Fiorentino, Italy) and 2 with glargine insulin (Lantus, Sanofi‐Aventis US LLC, Bridgewater, NJ). All dogs received twice daily insulin administration and all dogs received the same dose morning and evening. Median insulin dose was 0.56 U/kg (0.32‐1.62). Six of 20 dogs had concurrent disease; 3 dogs had pituitary dependent hypercortisolism and were receiving trilostane (Vetoryl, Dechra Pharmaceuticals, Northwich, England); 3 dogs had primary hypothyroidism and were receiving levothyroxine (Canitroid, Dechra Pharmaceuticals, Northwich, England). One dog was on enalapril (Enacard, Merial, Milan, Italy) and amlodipine (Amodip, Ceva Salute Animale, Agrate Biranza, Italy) treatment for hypertension and proteinuria.

All sensors reported IG concentrations within 60 minutes after application. In 8/20 dogs the sensor recorded for 14 days while in 12/20 dogs the sensor stopped recording IG before 14 days due to accidental detachment (4/20) or because the hand‐held reader showed persistently “LO” or “ERR” (8/20). In these dogs, the recording period of the sensor was 13 days in 2/20, 11 days in 2/20, 10 days in 4/20, 6 days in 1/20, 4 days in 1/20, and 2 days in 1/20. The median wearing period was 12 days (2‐14).

At the end of the wearing period, 3/20 dogs showed mild erythema at the site of application of the sensor but was self‐limiting and did not require specific treatment.

One hundred and twenty‐eight day‐time GCs were obtained: 42/128 IGCs were recorded at home using FGMS. 86/128 GCs were performed in the hospital of which 43/128 with FGMS and 43/128 with PBGM.

## 4 ASSESSMENT OF IGCS AND BGCS

### 4.1 FGMS vs PBGM

When comparing GCs acquired using the FGMS and PBGM in the hospital, the insulin dosing recommendation would have been the same in 33/43 cases (77%) cases considering the percentage of values in the ideal range and in 34/43 cases (80%) considering the glycemic nadir. Weighted Cohen *K* coefficient, intraclass correlation coefficient, polychoric correlation coefficient as well as Fisher exact test *P*‐value relative to this comparison are reported in Table 3.

**TABLE 3:** Weighted Cohen *K* coefficient, intraclass correlation coefficient, polychoric correlation coefficient, and Fisher exact test *P*‐value of the concordance analysis

	FMGS‐PBGM			Day‐day (home)			Home‐hospital		
	Estimate	Lower	Upper	Estimate	Lower	Upper	Estimate	Lower	Upper
Weighted Cohen *K*	.79	.72	.86	.20	−.29	.69	.68	.60	.80
Intraclass correlation	.88	.79	.84	.36	−.74	.92	.82	.59	.92
Polychoric correlation	.83	.64	.89	.29	−.25	.64	.67	.58	.89
Fisher exact test (*P*‐value)	.99	/	/	.89	/	/	.90	/	/

Abbreviations: FGMS, flash glucose monitoring system; PBGM, portable blood glucose meter.

### 4.2 Day‐day same environment (home)

When comparing IGCs performed on 2 consecutive days with the same device (FGMS) in the same environment (home) insulin dosing recommendation would have only been the same in 5/14 cases (36%) considering the percentage of values in the ideal range and in 9/14 cases (64%) considering the glycemic nadir. Weighted Cohen *K* coefficient, intraclass correlation coefficient, polychoric correlation coefficient as well as Fisher exact test *P*‐value relative to this comparison are reported in Table 3.

### 4.3 Different environment (home‐hospital)

When comparing IGCs performed on 2 consecutive days using the same device (FGMS) in 2 different environments (home and hospital) the insulin dosing recommendation would have been the same in 17/25 cases (68%) when considering the percentage of glucose measurements that were within the ideal range and in 16/25 cases (64%) considering the glycemic nadir. Weighted Cohen *K* coefficient, intraclass correlation coefficient, polychoric correlation coefficient as well as Fisher exact test *P*‐value relative to this comparison are reported in Table 3.

### 4.4 Assessment of nadirs

The AGP was used as a “gold‐standard,” with glucose nadirs identified in 34/43 (79%) IGCs. Nadirs could not be extrapolated from the AGP in 5/43 (12%) IGCs because the majority of IG measurements were above 350 mg/dL and so were not available, and in 4/43 (9%) owing to a reading error. In comparison with this “gold standard” and considering only the 34 IGCs in which a glucose nadir was identified, concordant glucose nadirs were detected in 28/34 (82%) IGCs generated by the individual FGMS scans and in 14/34 (41%) BGCs obtained by PBGM. The reasons why glucose nadirs were not detected by PBGM were: in 7/34 (21%) PBGM nadirs fell between 2 consecutive PBGM measurements, in 7/34 (21%) PBGM nadirs fell in a different time period than the nadir extracted from the AGP, and in 6/34 (18%) the nadir occurred after the hospitalization period.

### 4.5 Comparison between day‐time and night‐time nadir (study aim 4)

One hundred and fifty‐two, paired, day‐time and night‐time nadirs as recorded on the AGP software were available for analysis. Day‐time and night‐time nadirs were within the same glycemic range in 82/152 (55%) cases. The night‐time nadir was greater than day‐time nadir in 46/152 (30%) cases whereas the night‐time nadir was lower than day‐time nadir in 24/152 (15%) cases.

The median day‐time nadir was 147 mg/dL (40‐470 mg/dL) and the median night‐time nadir was 170 mg/dL (40‐351 mg/dL) (*P* = .02).

### 4.6 Assessments of low IG/hypoglycemic episodes

Using any method of measurement, a total of 66 low IG/hypoglycemic episodes were recorded in 13/20 (65%) dogs during the recording period however none of these dogs had signs suggestive of hypoglycemia. Analyzing the AGP, 66/66 low IG episodes were identified whereas using the individual FGMS scans 40/66 (60%) low IG episodes were detected. During the hospitalization period, the PBGM identified 6/66 (9%) hypoglycemic episodes in 5/20 dogs. All low IG/hypoglycemic episodes detected by the PBGM were also detected by the FGMS scans.

## 5 DISCUSSION

The main aim of the present study was to compare the clinical utility of FGMS with the traditional BGCs in the monitoring of dogs with DM. Overall, our results suggest that use of traditional BGCs as a clinical tool for dogs with DM has important limitations, especially for the detection of the glucose nadir, low glucose events, and day‐to‐day glycemic variability (GV). In comparison, FGMS appears to have greater clinical utility.

All dogs tolerated the use of a bandage to maintain the position of the FGMS and mild erythema was noted at the site of the sensor in only 3/20 (15%) dogs. The incidence of erythema at the site of application was much lower than the 50% reported in a previous study of dogs.^11^ Erythema occurs in 4% to 44% human patients with diabetes mellitus.^18^, ^19^, ^20^, ^21^ One potential cause for the cutaneous erythema is a dermatological reaction to the isobornyl acrylate that is contained in the sensor itself and that can migrate into the adhesive part of the device and come into contact with the skin.^22^ The mild erythema does not seem to create discomfort for the animal and can be considered a mild and acceptable side effect in the use of the FGMS.

One of the aims of this study was to compare the GCs generated using the FGMS with those simultaneously generated using a PBGM. Following the common interpretation of the indices, the correlation showed an optimal concordance between FGMS and PBGM generated GCs. Indeed, analysis of the GCs obtained with the use of FGMS and PBGM in the hospital showed that FGMS led to the same insulin dose recommendation in more than 75% of cases. In the majority of remaining cases the insulin dose deduced from the FGMS profiles was higher than those looking at the PBGM profiles. This most likely reflects the fact that the PBGM used in this study has been previously shown to underestimate the blood glucose concentration throughout all values of BG compared to the hexokinase method,^14^ whereas FGMS appears to only do so when BG is in the hypoglycemic range.^11^ Furthermore, the FGMS measures IG which, although shown to accurately estimate plasma glucose in humans, has been previously shown to have a wide time lag of 4 to 50 minutes when compared to circulating blood glucose.^23^ The time lag between plasma and IG appears to differ depending on whether plasma glucose values are rising or falling,^24^, ^25^, ^26^ the type of CGM instrument used as well as the sensor algorithm used.^27^, ^28^ In a previous veterinary study, the FGMS was unable to measure the rapid changes between the peripheral glucose and interstitial fluid glucose after the injection of a bolus of dextrose IV, although the median time lag was not reported.^11^ It is possible that the lag phase could have influenced the discrepancy between the recommended insulin dose from the 2 devices.

When analyzing subsequent day, home‐home, FGMS recorded IGCs, the same insulin dose recommendation was obtained in 36% of cases considering the percentage of values in the ideal range and in 64% of cases considering the glucose nadir. Following the common interpretation of the indices, the correlation showed an almost absent concordance between IGCs obtained on 2 consecutive days at home. Although Fisher exact test registered no significant difference (probably due to the low number of cases) this represent a clinically important difference. To the authors knowledge, there are no studies that have looked at this aspect in a home environment. Indeed, in a previous study looking at day‐to‐day variability of BGCs in diabetic dogs, dogs were maintained in the hospital during the entire monitoring period. In this study an opposite or different but not opposite theoretical recommendation for adjustment of the dog's insulin dose on day 2, compared with day 1, was made on 27% and 17% of occasions respectively, although individual insulin dose and meal were kept constant.^3^ Between‐day GV is also reported in 93% of human patients with DM^4^ and has been associated with daily fluctuations in the postprandial glycemic response to a standard meal,^29^variable sensitivity to insulin^30^ and variation in the rate of absorption of insulin from the SC injection site, particularly if different anatomic region are used.^31^ Additional factors include the degree of diabetic instability,^32^, ^33^, ^34^ the amount of residual β‐cell function^35^, ^36^ and inherent error associated with measuring insulin volume in a syringe.^37^ All of these factors could also be expected to influence blood glucose concentrations in diabetic dogs. Moreover, in human diabetic patients, long acting insulin preparations are associated with a reduced day‐to‐day GV compared to intermediate‐acting insulins.^38^ Hence, the different type of insulin used in this study might also have affected the day‐to‐day variations in glycemic control detected. Also, 6 dogs in this study had concurrent endocrine diseases known to cause insulin‐resistance and for which they were receiving medications. Variability in the intestinal absorption of these medications could partly explain the day‐to‐day variability of BGCs here detected. On the basis of the results of this current study, it seems that the reproducibility of glucose curves produced at home is not better than for those produced in a hospital environments and highlights the importance of performing and analyzing serial BGCs before making specific treatment decision or consider the use of CGMS that allow the monitoring of glucose concentrations during consecutive days.

Compared to subsequent day home‐home IGCs, when analyzing subsequent day, home‐hospital, FGMS recorded B IGCs, the same insulin dose recommendation was obtained in 68% of cases considering the percentage of values in the ideal range and in 64% of cases considering the glucose nadir. Using the common interpretation of the indices, the correlation showed a good concordance between IGCs conducted in the 2 environments on 2 consecutive days. The objective of this analysis was to evaluate if day‐to‐day variability between the home and clinic curves existed. As already above mentioned, in dogs with DM, serial BGCs performed in a hospital environment showed important day‐to‐day variability with concordant insulin recommendations in only 57% of cases.^3^ In that study, dogs received the same dose of porcine lente insulin every 12 hours and were fed at the time of insulin injection with the same nutritional balanced canned dog food divided into 2 equal‐sized meals each day. Despite this, there was marked disparity between the theoretical recommendations for dose adjustments based on the curves obtained on consecutive days, particularly in dogs with lower minimum blood glucose concentrations, which represented those with better glycemic control. This has important clinical implications, especially in well‐controlled dogs in which the increase in insulin dose can have serious sequelae. However, the dogs were maintained in a constant hospital environment. Differences in the feeding schedules and in the amount of exercise of the diabetic pet, as well as stress due to unfamiliar environment or repeated vein punctures could have contributed to that findings. In our study, insulin dose recommended from the FGMS profiles obtained in the hospital setting were higher than those obtained at home in approximately 50% of cases. These results are in line with a previous study looking at the treatment decision based on home and hospital generated BGCs in dogs with DM. In that study, the mean and maximum glucose concentration of the hospital curves were significantly lower than those of the home curves.^39^ A possible explanation for this finding is that blood glucose concentration in the clinic might be lower than those at home because of reduce appetite^39^ and thus reduced postprandial hyperglycemia. However, in our study dogs were fed at home before the admission to the hospital, therefore it is unlikely that the food consumption would have influenced this result. Also, stress hyperglycemia, which can lead to higher BGC values, might be a problem in cats but it is less frequently identified in dogs with DM.^1^

**FIGURE 2:**
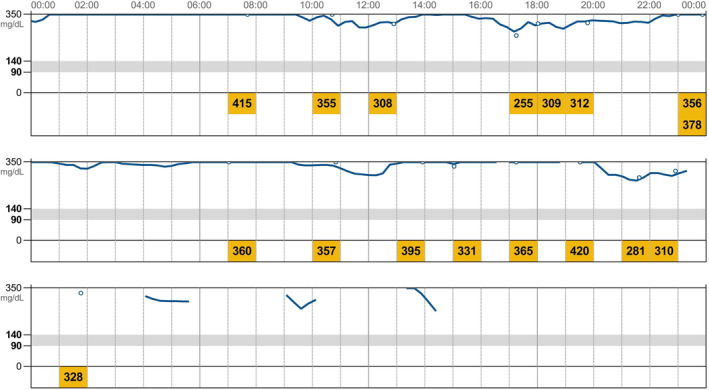
Daily log report showing the majority of IG fluctuations >350 mg/dL and gap in the graphs. Above 350 mg/dL the graphs are not generated, and no information can be extrapolated. IG, interstitial glucose

The AGP report was used as a “gold standard” and identified the glycemic nadir in 79% of IGCs. In 12% of cases, the glucose nadirs could not be extrapolated from the AGP because the IG readings were higher than 350 mg/dL and were thus not showed by the generated graphs (Figure 2). Additionally, in 9% of cases it was not possible to analyze the graph because it was not correctly generated owing gaps in the graph which could be the result of intermittent sensor dysfunction. In comparison with those AGP reports in which glucose nadirs were identified, concordant glucose nadirs were detected in 82% of IGCs obtained by individual FGMS scans and in only 41% of BGCs obtained by PBGM. In the majority of cases this was due to the nadir either falling in a different time period than the nadir extracted from the AGP or after the period of hospitalization. As the nadir is crucial for determining the appropriate insulin dose, the use of FGMS that measures IG levels every minute likely provides more information regarding glucose patterns and trends, potentially allowing a more correct dose recommendation. However, in those dogs in which the hand‐held reader is not able to display the glucose concentrations (because of the reading errors), the use of a PBGM might be useful to confirm the true BG value.

Comparison between the day and night‐time nadirs showed that in more than half of the cases, the nadirs were in a different glycemic range. The majority of night‐time nadirs that were in a different glycemic range were higher than the day‐time nadirs. This was highlighted by the difference between the median night‐time and day‐time nadirs (170 and 147 mg/dL, respectively). A previous study, in which a different CGMS was used, found no difference between day and night‐time mean, maximum and minimum glucose concentrations. However, the dogs in the aforementioned study were maintained under controlled living conditions including room temperature, humidity and light/dark cycle limiting the influence of environmental factors.^10^ In contrast, dogs in our study were maintained in their normal environment where variation in environmental might have had a greater impact on variation in glycemic control. Circadian hormone secretory patterns might also affect glucose fluctuations in diabetic dogs, although in this species, significant circadian secretory fluctuations of the major counterregulatory hormones such as cortisol and growth hormone have not been demonstrated^40^, ^41^ and, as such, weakly, if at all, affected glucose fluctuations in the present study. In human medicine the opposite is observed; hypoglycemic episodes have been shown to be more common at night compared with during the day.^42^, ^43^ The risk of nocturnal hypoglycemia in human diabetic patients is associated with various factors including age, insulin dose, site of injection, temperature and day‐to‐day intraindividual variation in the rate of insulin absorption which might vary up to 50%.^44^ All dogs in this study and in the aforementioned veterinary study, were fed the same amount of food and were injected with the same doses of insulin twice daily at a 12‐hours interval. Thus, daily glucose fluctuations might be lower in dogs than in humans who regularly eat 3 meals each day and inject insulin 3 or 4 times each day.^10^ Additionally, during the day dogs might perform more physical activity than during the night. It is well established that exercise causes increases in insulin‐stimulated whole body glucose disappearance, muscle glucose uptake, and muscle nonoxidative glucose metabolism^45^ thus making daily glucose fluctuations lower during the day.

During the study period, 65% of dogs exhibited low IG/hypoglycemic episodes but no clinical signs of hypoglycemia were documented. The FGMS individual measurements and PBGM allowed identification of 60% and 9% of the low IG/hypoglycemic episodes, respectively. Hypoglycemia is a potentially serious complication in insulin‐treated humans and dogs with DM.^1^, ^43^ Hypoglycemic episodes can be easily missed when using a PBGM owing to the logistical difficulty of frequent blood sampling. Moreover, decreases in blood glucose can be followed by hyperglycemia or viceversa. In humans diabetology, glycemic excursion consisting of episodes of hypoglycemia followed by hyperglycemia or of hyperglycemia followed by hypoglycemia, with no apparent causal link, is defined as GV.^46^ Although the concept of GV has not yet been investigated in dogs, in humans this concept gained particular attention in recent years and it is emerging as an additional glycemic target.^47^

If a PBGM is used, important glucose fluctuations might be missed between BG measurements and can result in erroneous insulin dose recommendations.^15^, ^48^ Given the that the FGMS detects a greater number of low IG episodes it could be the preferred choice for monitoring of DM in dogs. In human medicine the use of FGMS significantly reduces duration and frequency of hypoglycemia in patients with type 1 and type 2 DM^49^, ^50^, ^51^, ^52^ as well as GV.^53^ This effect is likely due to a combination of on‐demand access to real‐time sensor glucose results with trend arrows, enabling preventive action and informing behavior modification to alter the balance of insulins.^50^ The clinical accuracy of the FGMS has been demonstrated in dogs previously; however, a decreased in accuracy was noted at BG values less than 70 mg/dL with 69% of IG measurements underestimating peripheral BG measurements by an average of 15.4 mg/dL.^11^ Despite this, the FGMS was used as the gold standard for detection of hypoglycemia in this study owing to a greater recording time which, in theory, would reduce the likelihood of “missing” episodes of biochemical hypoglycemia. Considering all the above, FGMS can be a valuable tool in detecting low IG episodes. Furthermore, its usefulness is not limited to the retrospective analysis of the graphs generated by the AGP software, as real‐time IG concentrations can be obtained on a minute by minute basis by scanning the sensor with the hand‐held reader. A glucose trend arrow (indicating rate and direction of change in glucose levels) and a graphical trace of glucose values for the previous 8‐hour period are also displayed on the screen.

Some of the limitations of the FGMS are related to the fact that the sensor is designed for human patients with DM where stricter glycemic control is attempted. For example, the graphs generated by the AGP show glycemic values up to 350 mg/dL and above this, values are not reported (Figure 2) unless the sensor is scanned by the reader. This aspect can limit the utility of the sensor in dogs with poorly controlled DM. Finally, to obtain continuous IG measurements the sensor needs to be scanned at least every 8 hours, which cannot always be logistically possible.

There were a number of limitations in the present study. First, only a small number of dogs were included in the study. Second, in the majority of the dogs the sensor lasted less than 14 days, thus limiting the number of data available for the analysis. Third, we did not evaluate if the accuracy of the system varies during the entire wearing period. However, some studies from human medicine demonstrate that accuracy of the device remain stable through 14 days of use.^18^, ^20^, ^54^, ^55^ Also, in the previous study in which FGMS was used in diabetic dogs mean absolute relative difference (MARD) on 14th day was only slightly higher compared to the MARD on 1st day.^11^ Finally, considering the absence of a gold standard as a monitoring tool for diabetic dogs, in this study FGMS has been used as the “gold standard” to compare its ability in detecting low glucose measurements and glucose nadirs compared to the traditional BGCs because it allows a 24 hours recording time, even if in the hypoglycemic range it is less accurate.

## CONFLICT OF INTEREST DECLARATION

Authors declare no conflict of interest.

## OFF‐LABEL ANTIMICROBIAL DECLARATION

Authors declare no off‐label use of antimicrobials.

## INSTITUTIONAL ANIMAL CARE AND USE COMMITTEE (IACUC) OR OTHER APPROVAL DECLARATION

The study was approved by the Scientific Ethical Committee for Animal Testing of the Alma Mater Studiorum ‐ University of Bologna.

## HUMAN ETHICS APPROVAL DECLARATION

Authors declare human ethics approval was not needed for this study.
